# Indicator Tubes: A Novel Solution for Monitoring Temperature Excursions in Biobank Storage

**DOI:** 10.3390/mps8050120

**Published:** 2025-10-03

**Authors:** Patrick J. Catterson, Tyler T. Olson, Margaret B. Penno, Steven P. Callahan, Melissa V. Olson

**Affiliations:** The Johns Hopkins BioBank, Genetic Resources Core Facility, Department of Genetic Medicine, Johns Hopkins University School of Medicine, 600 N. Wolfe St., 1017 Blalock, Baltimore, MD 21287, USA

**Keywords:** biobanking, specimen integrity, cellular viability, long-term cryogenic storage, biobanking quality assurance, temperature excursion detection, temperature indicators

## Abstract

Maintaining the integrity of cryogenically preserved biological materials is critical, as even brief, undetected temperature excursions in storage can compromise sample viability. Existing monitoring systems may miss transient thaw–refreeze events, posing serious quality risks. To address this, we developed and validated frozen indicator tubes that visually signal deviations from the frozen state, serving as a cost-effective backup to electronic monitors. Our first method uses an aqueous dye solution that immobilizes the dye when frozen; any thawing causes the dye to disperse, providing a clear, external visual cue of a partial or complete thaw. For ultra-low-temperature storage (−80 °C), we introduced a second method using an ethanol-based solution calibrated to indicate thaw events. This system detects temperature rises of 10 °C or more sustained for at least fifteen minutes—conditions that may jeopardize sample stability. When paired with standard monitoring systems, these indicator tubes offer an added layer of protection by providing simple, reliable, and immediate visual confirmation of critical temperature breaches. This innovation enhances confidence in cryogenic storage protocols and supports the long-term preservation of sensitive biological materials.

## 1. Introduction

The preservation of biological materials in a frozen state is fundamental to both biomedical research [1,2,3,4], as well as clinical [3,5] and therapeutic [6,7,8] applications. The viability of these materials critically depends on maintaining low temperatures, as even minor deviations can precipitate irreversible damage [9,10,11]. Despite their importance, contemporary temperature monitoring systems frequently fail to detect brief yet significant partial thaw–refreeze events directly to specimens, presenting a critical vulnerability in cryopreservation protocols.

Detecting subtle partial thawing events is crucial, as these can compromise sample integrity without triggering alarms configured for complete thaw scenarios. Brief exposure to elevated temperatures can cause microscopic melting and compromise viability [11] and biological integrity [8,12]. Lower storage temperatures are often considered ideal for biologics, as they increase sample stability and preservation duration [13]. Cryogenic storage in liquid nitrogen is standard for preserving long-term viable cells, tissues, and organisms, which can remain viable for extended periods under stable conditions [10]. Ultra-low-temperature storage at −80 °C is critical for the stability of biologics used in rapid workflows, research analytes, therapeutics, non-viable biologics, and many secondary components of viable tissues and cells [14].

Current frozen status monitoring methods primarily rely on electronic sensors and data loggers, which continuously record storage temperatures, provide alarms, and generate compliance documentation [14,15]. While effective for detecting gross deviations, these systems depend heavily on probe placement and may fail to capture localized or transient thermal fluctuations at the specimen level. Complementary chemical and physical threshold indicators, such as FreezeWatch^®^ or TempTracker^®^, are also used to provide a visual cue when a predefined temperature has been exceeded. However, these devices are generally external to the sample, are single-use, short-term, and do not reliably detect partial thawing or refreezing events. Cryostorage facilities further employ tank-level monitoring of liquid nitrogen volume or vapor-phase stability, which helps ensure overall temperature control but cannot rule out localized thaw events during handling, shipment, or tank failure [15]. Taken together, these approaches highlight the need for supplementary, specimen-level monitoring tools to safeguard sample integrity.

To mitigate these challenges, we have developed a novel solution that enhances existing electronic monitoring systems as a secondary component to best practices in modern biobanking [15]. Our frozen indicator tubes provide an unmistakable visual cue that signals temperature deviations potentially jeopardizing sample integrity in a low-temperature state. This dual monitoring strategy integrates continuous electronic oversight with a fail-safe, physical indicator, thereby enhancing confidence in the integrity of biological materials during frozen-state storage.

## 2. Experimental Design

This protocol describes the development and evaluation of two cryogenic indicator vial systems designed to detect partial thaw events under different temperature conditions: standard frozen storage at −20 °C and ultra-low-temperature storage at −80 °C. The goal is to provide a simple, visual cue indicating potentially harmful temperature excursions, supplementing standard electronic monitoring systems. The −20 °C indicator relies on dye dispersion from water-soluble food-grade sprinkles, while the −80 °C version uses Quinaldine Red powder and an ethanol-based solution for sensitive detection of partial thawing. Controlled thaw/refreeze tests were conducted to validate the functionality of each system.

### 2.1. Materials

- Thermo Scientific™, Nunc™, Biobanking and Cell Culture Cryogenic Tubes, 1.8 mL (Thermo Fisher Scientific, Waltham, MA, USA; Cat. no.: 375418).
- Thermo Scientific™, Cryo Vial Closure Color Coders, red (Thermo Fisher Scientific, Waltham, MA, USA; Cat. no.: 375515).
- Food-grade nonpareil sprinkles (various commercial suppliers).
- Quinaldine Red (Thermo Fisher Scientific, Waltham, MA, USA; Cat. no.: AC197410010).
- Distilled water.
- Ethanol (Warner-Graham Company, Cockeysville, MD, USA; Cat. no.: 64-17-5).
- KimTech Science™, Precision Wipes (Kimberly-Clark, Irving, TX, USA; Cat. no.: 34155).
- Pencil, scissors, and forceps.
- Thermosafe multi-purpose insulated shipper: 355 EPS with corrugated carton, 1.5″ Wall Thickness, 36″ × 36″ × 37″ (Sonoco, Hartsville, SC, USA; Cat. no.: 03-530-17).
- Dry Ice.

### 2.2. Equipment

- A −20 °C freezer (LabRepco, Horsham, PA, USA), a −80 °C ultra-low-temperature freezer (Panasonic PHC, Tokyo, Japan), a vapor-phase liquid nitrogen storage unit (MVE), and a controlled-rate freezer CBS 2100 (Custom Biogenics, Bruce Township, MI, USA), all electronically monitored for temperature excursions and mapped for consistent temperature throughout the unit.
- A temperature monitoring probe (Rees Scientific, Trenton, NJ, USA).
- A liquid nitrogen dewar.
- A laboratory balance (0.1 mg precision).

## 3. Procedure

**Figure 1:**
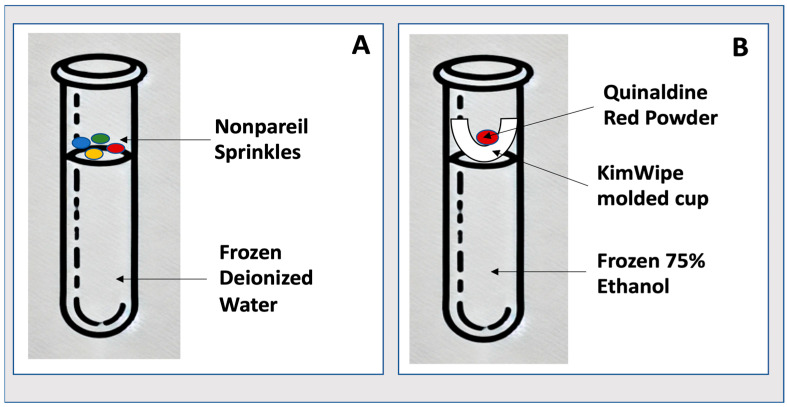
Indicator models. Depending on the cryopreservation need, indicators were modeled to detect partial thawing following storage in a frozen environment (−20 °C) (**A**) and in an ultra-low-temperature environment (−80 °C) (**B**).

The following procedures describe step-by-step instructions for constructing and validating frozen indicator vials designed for use at both −20 °C (Figure 1A) and −80 °C (Figure 1B). Each method includes critical steps for proper assembly and visual inspection, allowing researchers to adapt the indicators to their specific storage environments.

### 3.1. Making the Frozen Storage Indicator (−20 °C)

**Figure 2:**
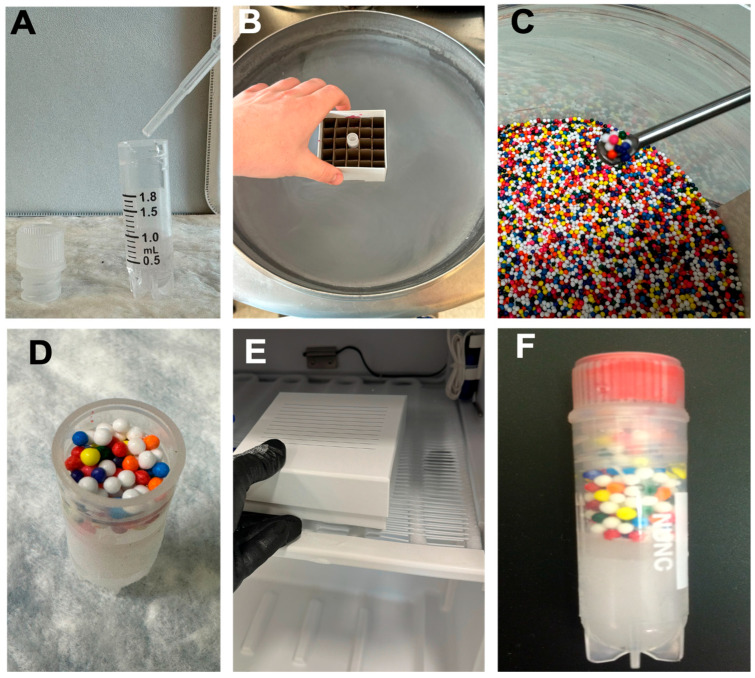
Indicator methods in the frozen environment (−20 °C). Frozen indicators were generated by first freezing deionized water (**A**,**B**), adding sprinkles atop the ice (**C**,**D**), and preserving at −20 °C (**E**,**F**).

The time required is ~24 h, including freeze and inspection. The development procedure is outlined in Figure 2.

1. Fill each cryogenic vial with 1 mL of deionized water.
2. Place vials in a −20 °C freezer until completely frozen (minimally overnight).
3. Once frozen, open the vial and gently add ~0.8 g of food-grade sprinkles to the ice surface. CRITICAL STEP: Ensure sprinkles rest on the surface without embedding into the ice.
4. Cap the vials and return to their designated position in frozen storage.
5. Inspect regularly. If sprinkles remain intact, no thaw has occurred. If dispersed, thaw has occurred.

### 3.2. Making the Ultra-Low-Temperature Storage Indicator (−80 °C)

**Figure 3:**
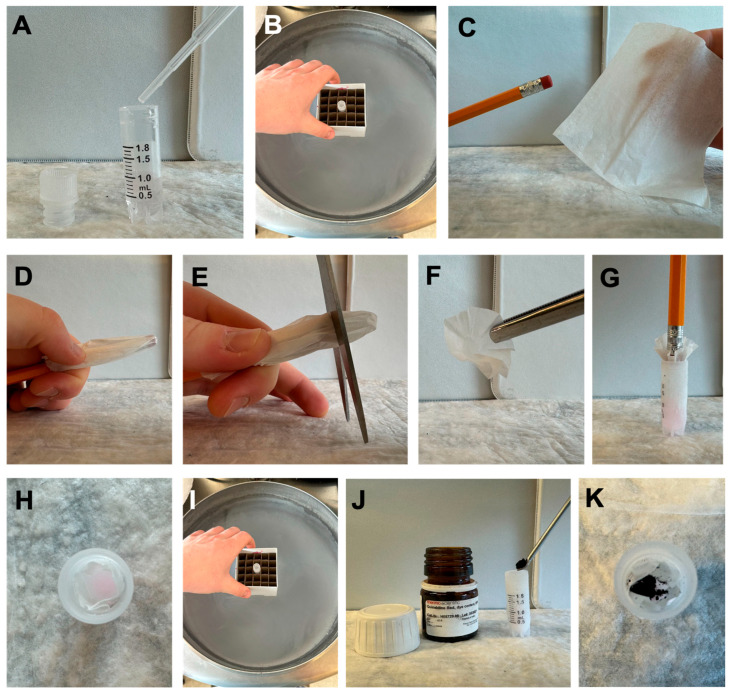
Frozen indicator methods in the ultra-low-temperature environment (−80 °C). Frozen indicators were generated by freezing a 75% ethanol solution (**A**,**B**), generating a ‘cup’ (**C**–**H**), chilling (**I**), and then adding indicator dye (**J**,**K**).

The time required is ~2 h, including preparation and freezing. The development procedure is outlined in Figure 3.

1. Add 1 mL of 75% ethanol (deionized water and ethanol) to a cryogenic vial.
2. Freeze the vial in a liquid nitrogen vapor-phase freezer to solidify the ethanol (minimally one hour).
3. Cut a small 1-inch square from a KimTech wipe and form a cone using a pencil.
4. Form a cone shape with the cut square around a pencil. CRITICAL STEP: Handle gently to avoid tearing, which could compromise the indicator’s functionality.
5. Slide the cone approximately two centimeters off the pencil and trim the end to create a ‘cup’.
6. Insert the cup into the vial, resting on top of the frozen solution.
7. Chill the entire assembly under a liquid nitrogen vapor phase for 5 min.
8. Add ~35 mg of Quinaldine Red powder into the cup and cap the vial.
9. Place the vial in −80 °C storage.
10. Inspect regularly. Dye dispersion (pink in ethanol) signals partial thawing.

### 3.3. Validation Tests

**Figure 4:**
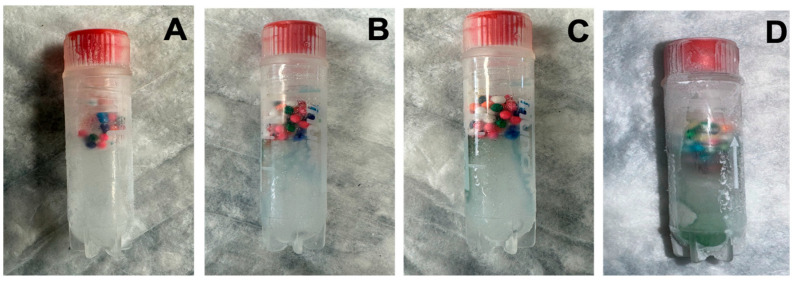
Frozen vial validation (−20 °C). Indicators were frozen and maintained for more than 1 h at −20 °C (**A**), then thawed at room temperature for 5 and 8 min, respectively (**B**,**C**), and then refrozen at −20 °C (**D**).

Proof-of-principle tests were performed to ensure indicator vials were capable of detecting partial thaw events. As such, frozen indicators (Figure 4), stored at −20 °C, were removed from the freezer and exposed to ambient room temperature (~22 °C) for defined intervals of 5 min and 8 min. After each exposure period, the vials were returned to −20 °C storage. Following refreezing, the vials were examined to evaluate sprinkle dye dispersion as a measure of thaw detection.

**Figure 5:**
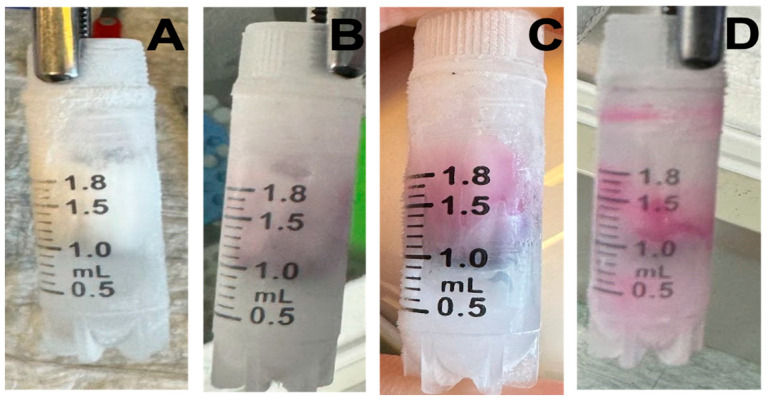
Ultra-low-temperature vial validation (−80 °C). Ultra-low-temperature indicators were frozen and maintained for 1 h at −80 °C (**A**), then thawed in a controlled-rate freezer at −60 °C for 30 and 60 min, respectively (**B**,**C**), and then refrozen at −80 °C (**D**).

For the ultra-low-temperature vial test (Figure 5), ethanol-based indicator vials stored at −80 °C were removed and placed in a controlled-rate freezer at −60 °C for defined intervals of 30 min and 60 min. After each exposure period, the vials were returned to −80 °C storage. Following refreezing, the vials were examined to evaluate dye dispersal as a measure of thaw detection.

**Figure 6:**
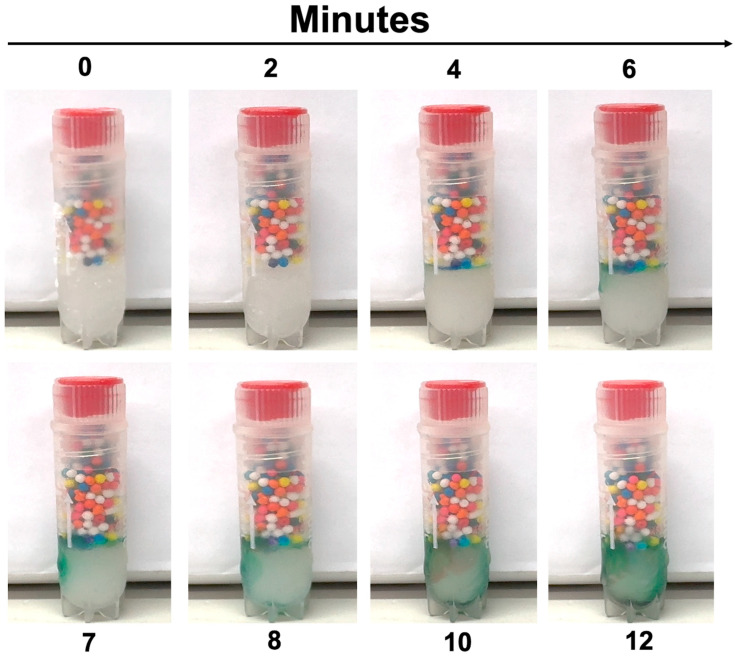
Partial thaw detection for the frozen indicator. Vials were thawed over time in a room-temperature environment. Within minutes, a partial thaw was detected by an increase in dye intensity and enhanced over time.

**Figure 7:**
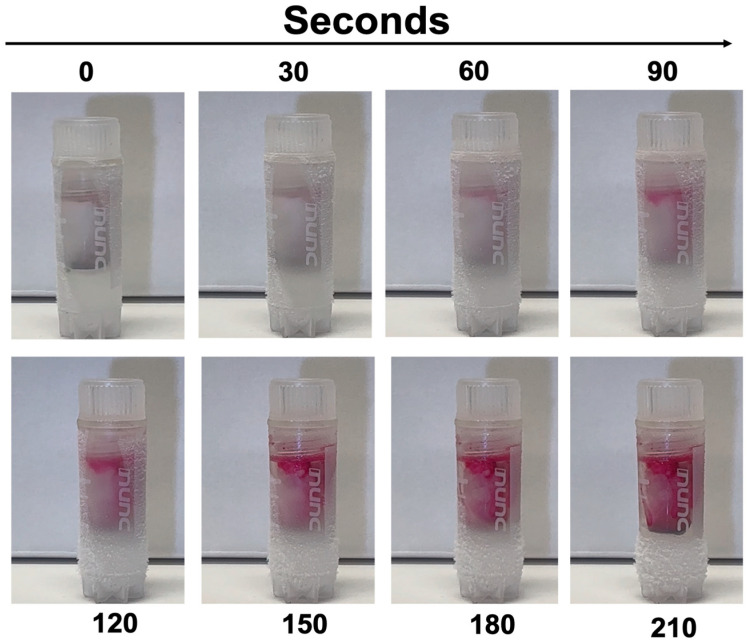
Partial thaw detection for the ultra-low-temperature indicator. Vials were thawed over time in a room-temperature environment. Within seconds, a partial thaw was detected by an increase in dye intensity and enhanced over time.

To confirm that dye bleed correlates with partial thaw duration, we further validated frozen indicators that were subjected to room temperature from 0 to 12 min (Figure 6) and from 0 to 210 s for ultra-low-temperature indicators (Figure 7). The gradual increase in darkening dye directly correlates with the increase in vial exposure to warm temperature, confirming a partial thawing event. This transition in color was irreversible and confirmed to be sustained when refrozen.

**Figure 8:**
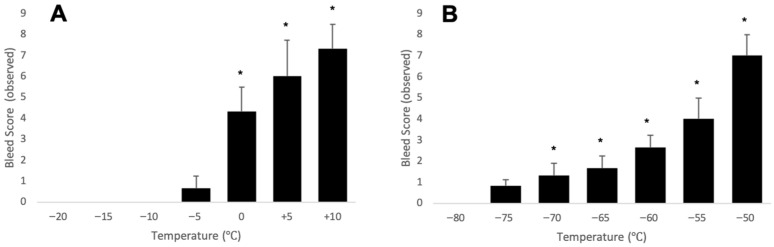
Partial thaw detection mapped. Vials were sustained for 15 min at various temperatures ranging from fully frozen (−20 or −80 °C) to temperatures that would induce a partial thaw event for frozen vials (**A**) and ultra-low-temperature vials (**B**). Using a visual bleed score (Supplementary Materials), a gradual increase in dye detection was seen as temperatures rose, with significant detection (*) at 0 °C and higher in the frozen indicators and −70 °C and higher in the ultra-low-temperature indicators.

After validating the visual indication of a dye upon partial melting, a scale was established from 0 to 10 as a ‘bleed score’, where 0 represented no spread of dye and 10 represented opacity and a dark dye (see the Supplementary Materials for further breakdown). This scale was used to assess frozen and ultra-low-temperature indicator status when vials were held in a controlled-rate freezer at temperatures ranging from fully frozen (−20 °C or −80 °C, respectively) to partial melting status for 15 min (Figure 8). Warming temperatures and thus partial freeze events correlated directly with an increasing bleed score.

### 3.4. Practical Application Testing

To evaluate real-world utility beyond stationary biobanking conditions, both frozen and ultra-low-temperature indicator vials were prepared for shipment simulation. Each vial was supplemented with an additional Kimwipe (1 in. × 1 in. square) positioned above the dye/sprinkles as a ‘packing material’ and placed in a ThermoSafe 355 EPS Foam multi-purpose insulated shipper container with a corrugated carton containing 10 lbs of dry ice. Dry ice, the solid form of carbon dioxide (CO_2_), maintained a temperature of approximately −75 °C within the insulated shipper as confirmed by electronic monitoring. The vials were subjected to repeated aggressive jostling and shaking events for a minimum of 5 min within the box, followed by placement in a temperature-mapped controlled-rate freezer for 15 min. Throughout these handling simulations, the frozen status remained intact, consistently demonstrating a bleed score of 0. Subsequent thaw testing confirmed that the vials performed as expected, with results significantly consistent with those shown in Figure 8.

In addition to shipment simulation, the stability of the indicator vials was assessed over extended storage periods. Indicator vials were prepared and maintained at fully frozen status (−20 °C for frozen vials and −80 °C for ultra-low-temperature vials) without disruption and observed periodically (approximately 1-week intervals) for any evidence of residual dye leakage. All freezers in which vials were stored were both temperature-monitored and freezer-mapped to ensure stable and consistent storage conditions. Frozen indicators were monitored for more than 5 years as a regular component of the quality assurance plan within our current biobanking processes, and ultra-low-temperature indicators were monitored for 90 days. Over time, no dye dispersal or leakage was detected (a bleed score of 0), and vials performed as expected when exposed to partial thawing temperatures, confirming the long-term stability of the indicators under standard frozen and ultra-low-temperature storage conditions. 

## 4. Expected Results

The frozen and ultra-low-temperature indicator vials developed in this study provide a clear and immediate visual signal of partial thawing events, offering a valuable addition to cryopreservation quality assurance protocols. In both standard frozen (−20 °C) and ultra-low-temperature (−80 °C) conditions, thaw-induced changes were readily visible through the vial walls, enabling non-invasive, real-time detection of temperature excursions. The method is adaptable to a wide range of biobanking scenarios and frozen or cryogenic shipment containers.

In practice, partial and complete thawing triggered dispersion of food-grade sprinkles or Quinaldine Red across the frozen surface. These color changes correlated directly with thaw duration and remained visible after refreezing, enabling retrospective detection of events as brief as 6 min in frozen vials and as short as 60 s in ultra-low-temperature vials. Exposure to ambient conditions produced a gradual, observable color shift—from a compact dye pellet to a diffuse colored solution—clearly visible through the vial. Importantly, frozen indicators showed significant dye detection at 0 °C, while ultra-low-temperature indicators responded as early as −70 °C. As warming continued, color intensity increased until complete thaw and full vial opacity were reached.

Unlike commercial products such as TempTale^®^ or FreezeWatch^®^, which are designed primarily as external monitors for shipping and storage, our indicator vials are embedded directly into cryogenic sample containers. This enables specimen-level assessments of freeze–thaw integrity, rather than relying on external sensors that may not capture localized thawing events. While commercial systems provide reliable digital records, alarms, and logging capabilities, they are generally higher-cost solutions intended for large-scale shipments or short-term monitoring. In contrast, our vials offer an immediate, low-cost, visual readout of thawing events at the sample level. Long-term testing confirmed system stability, with frozen indicators remaining intact for more than five years and ultra-low-temperature models remaining stable for at least 90 days. This reliability makes them particularly advantageous for biobanking and clinical research settings, where large numbers of samples must be monitored routinely and integrity must be confirmed quickly and cost-effectively.

In considering the use of these indicator vials for more broad-reaching biobanking applications, we tested the potential for false negatives outside of stationary biobanking practices. If the dye pellet becomes dislodged and loses contact with the frozen surface, thawing may not result in a visible cue. To mitigate this, a packing material (Kimwipe) was positioned above the dye to maintain consistent contact. Stress testing—including repeated shaking and shipment simulation in insulated foam coolers packed with dry ice—demonstrated that this modification preserved reliability. Notably, no false positives were observed under these conditions.

Further testing confirmed the longevity and adaptability of the system. Frozen indicators remained stable for more than five years, and ultra-low-temperature vials maintained performance for at least 90 days under controlled storage. Worth noting in this model is that sensitivity can be tuned by adjusting ethanol concentration, allowing customization to specific biobank requirements. While additional work is needed to fully map alcohol concentration to temperature thresholds, this flexibility suggests potential future applications, including adaptation for vapor-phase liquid nitrogen (LN_2_) storage, thereby extending the utility of this proof-of-principle model. While not yet validated at <−130 °C, this approach could be extended to LN_2_ vapor-phase storage by modifying the solvent matrix or positioning indicators at handling interfaces. These indicators offer a proof-of-principle approach to a highly customizable biobanking temperature monitor device.

Finally, the choice of materials—food-grade sprinkles, 75% ethanol, and Quinaldine Red—ensured both safety and effectiveness. Ethanol’s reduced flammability at ultra-low temperatures and Quinaldine Red’s favorable safety profile make the system well-suited for routine laboratory use and shipment. 

The strength of this approach lies in its simplicity, safety, and sensitivity. Because the color change is irreversible and visible to the naked eye, these indicators can serve as a low-cost, low-maintenance backup to electronic monitoring systems. As a supplementary quality assurance measure, the indicators enhance confidence in frozen storage stability, offering an immediate and visual method to detect previously undetectable thaw–refreeze events.

## Data Availability

Data is available through author correspondence.

## Supplementary Materials

The following supporting information can be downloaded at: https://www.mdpi.com/article/10.3390/mps8050120/s1.
