# Supplementary material for: Indicator Tubes: A Novel Solution for Monitoring Temperature Excursions in Biobank Storage
Source: Methods Protoc. 2025 Oct 3;8(5):120. doi: 10.3390/mps8050120 (PMC12566185; doi:10.3390/mps8050120)

Visual Color Scales Frozen Indicators

reference scale of color bleed, numbered 0-10

Example:

0 = Clear / baseline, no visible dye spread (would look like an empty vial or clear matrix).

2–3 = Early bleed (faint, localized dye patches; mostly intact sprinkles).

4–5 = Moderate bleed (dye visibly diffusing, multicolored spread beginning).

6–7 = Strong bleed (colors more diffuse, mixture more obvious, boundaries lost).

8–10 = Intense / full melt (dark green-blackish hue, blended colors, strong opacity).

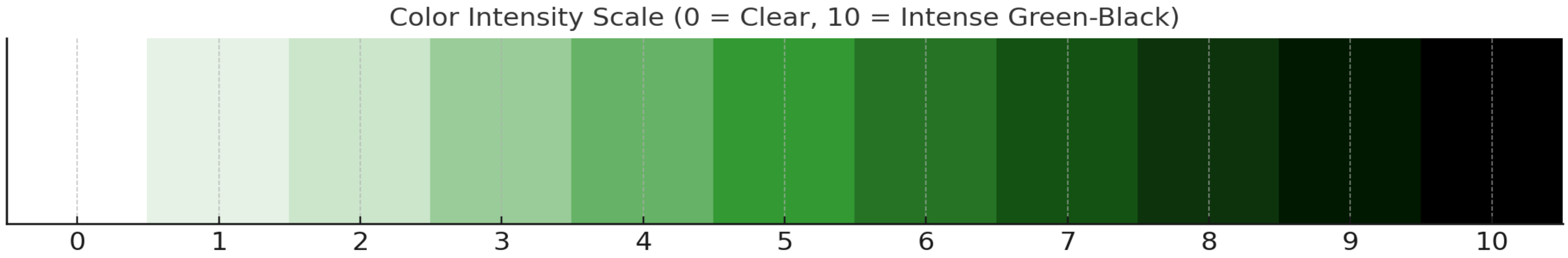

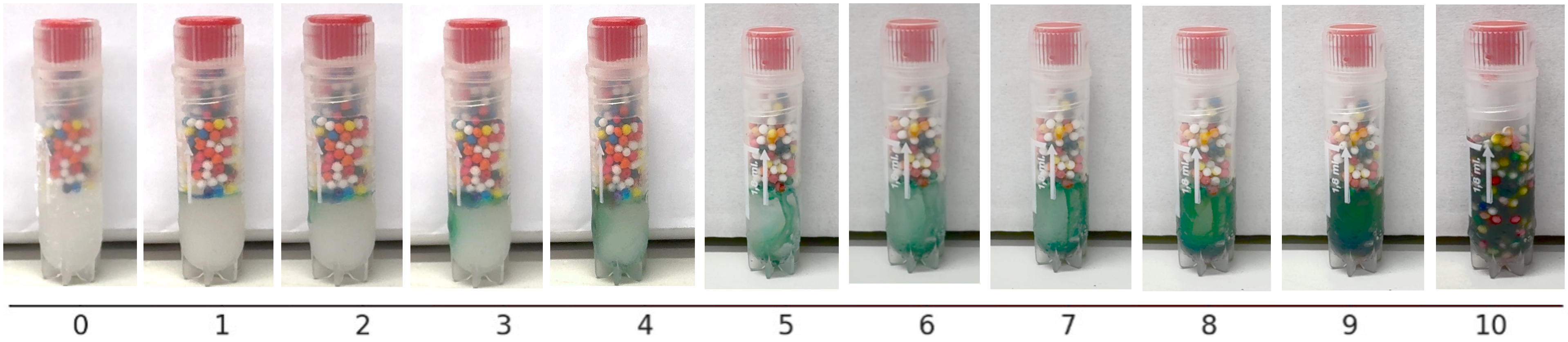

Visual Color Scales UltraCold Indicators

reference scale of pink-to-red shades, numbered 0–10

Example:
0 = clear
1–3 = pale pinks
4–6 = moderate rose/light red
7–9 = strong red
10 = deep, saturated red

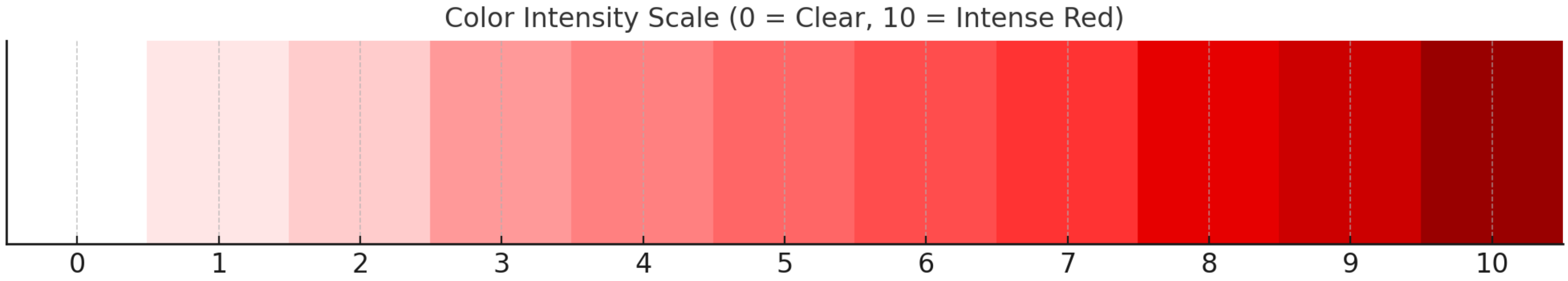

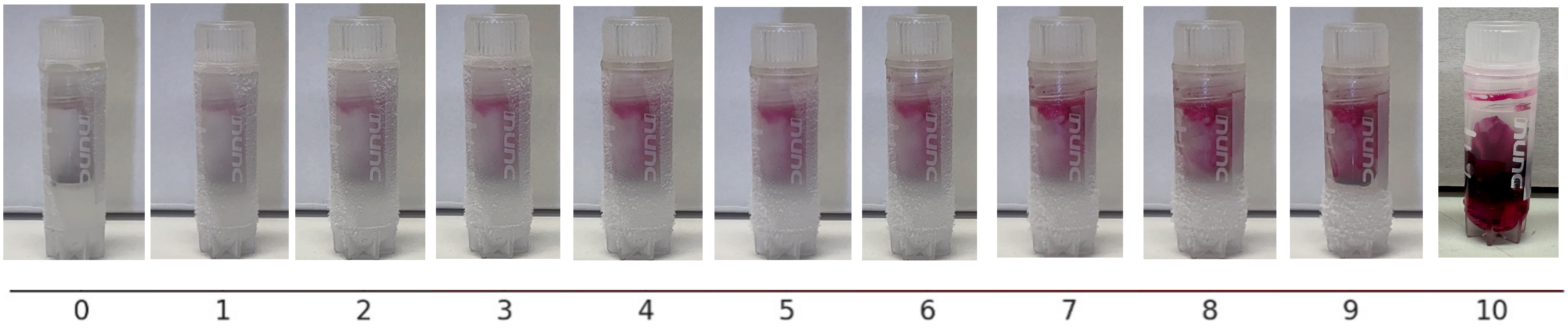

Supplement: Supplementary file 1 [file mps-08-00120-s001.zip › mps-3836083-supplementary.pdf]
